# A Rare Case of Congenital Glucose Galactose Malabsorption Due to SLC5A1 Mutation

**DOI:** 10.7759/cureus.98692

**Published:** 2025-12-08

**Authors:** Hema Muralidharan, Amreen Sajith, Nadia ElSayed, Juwairiya Syed Iqbaluddin, Poorvi Gupta, Dafalla Ahmed Babiker Rahamtalla

**Affiliations:** 1 Pediatrics, Al Qassimi Hospital for Women and Children, Sharjah, ARE; 2 Pediatric Gastroenterology, Al Qassimi Hospital for Women and Children, Sharjah, ARE

**Keywords:** acute kidney injury, fructose-based formula, glucose-galactose malabsorption, nephrocalcinosis (nc), osmotic diarrhea

## Abstract

Protracted neonatal diarrhea is severe and potentially life-threatening if not promptly diagnosed and treated. Causes include congenital defects in sodium, chloride, glucose/galactose, bile acid transport, enterokinase deficiency, and villous atrophy. Glucose-galactose malabsorption (GGM) is a rare autosomal recessive disorder caused by mutations in the *SGLT1* gene, impairing glucose and galactose absorption and leading to osmotic diarrhea. This case report highlights the rarity of GGM and emphasizes the importance of early recognition to enable prompt dietary intervention, preventing failure to thrive and reducing mortality. It further underscores the imperative need for fructose-based formulas to be readily available.

We describe a five-week-old full-term male infant, born to third-cousin parents, who presented in hypovolemic shock due to persistent diarrhea that started at two days of life, accompanied by severe metabolic acidosis and acute kidney injury. Abdominal ultrasound revealed medullary nephrocalcinosis. Endoscopic evaluation showed nonspecific duodenitis. The patient initially failed to reach full feeds on multiple formula trials, including amino acid-based, extensively hydrolyzed, and rice-based formulas, necessitating parenteral nutrition, which was later discontinued due to fungemia. Extensive investigations and genetic testing (whole exome sequencing) confirmed congenital GGM (SLC5A1 mutation). Due to the unavailability of glucose and galactose-free formula, he was initially started on a ketogenic formula and subsequently transitioned to a fructose-based formula once available. Following dietary modification, the patient showed significant clinical improvement, with resolution of diarrhea, steady weight gain, and appropriate growth on follow-up. In conclusion, GGM should be considered in infants with persistent diarrhea and failure to thrive. Timely diagnosis optimizes prognosis, improves outcomes, and facilitates genetic counseling for affected families.

## Introduction

Neonates are highly susceptible to diarrhea-related complications due to the immaturity of fluid homeostasis and immune regulation. Protracted diarrhea is characterized by persistent, severe symptoms that often necessitate parenteral nutritional support. Its etiology is diverse and can be classified based on villus-crypt architecture integrity, either preserved or disrupted with villus atrophy [1]. Well-documented causes include congenital defects in sodium, chloride, glucose/galactose, and bile acid transport, as well as congenital enterokinase deficiency, all of which contribute to early-onset severe diarrhea [2].

Congenital glucose-galactose malabsorption (CGGM) is a rare autosomal recessive disorder caused by mutations in the SLC5A1 gene, which encodes the sodium-dependent glucose transporter 1 (SGLT1). This results in defective absorption of glucose and galactose in the small intestine, thereby resulting in severe osmotic diarrhea and dehydration in neonates, which can be life-threatening if not diagnosed and managed early [1,2]. Due to the rarity of this disease, it is challenging for most clinicians to consider CGGM as an initial diagnosis. Identifying the underlying etiology is critical for prognosis, guiding therapeutic interventions, improving family and healthcare provider counseling, and assessing hereditary risk in affected families.

Here, we present the case of a term male newborn who was admitted to our hospital with severe protracted diarrhea. We have outlined the clinical course and comprehensive diagnostic workup performed for the patient, which ultimately led to the diagnosis of CGGM.

## Case presentation

A male baby, born at term to consanguineous parents, had an unremarkable antenatal and postnatal history. At two days of age, he developed episodes of loose, non-bloody, non-mucoid stools and was brought to the emergency department at four days of age, after which he was discharged with symptomatic management as he was vitally stable. At nine days of age, he was brought again due to vomiting that occurred mainly after feeds and was discharged after symptomatic management. He was on combined feeds of breastmilk and standard formula.

At five weeks of age, he was brought back to the emergency department with a two-day history of tactile fever, diarrhea (non-bloody, non-mucoid) that had been present for a month and had worsened over the last two days (occurring five to six times per day, watery, in large volume), along with decreased activity. He was admitted to the pediatric intensive care unit as a case of gastroenteritis with severe dehydration, hypovolemic shock, severe metabolic acidosis, electrolyte imbalance (hypokalemia, hypercalcemia, hypermagnesemia, and hypernatremia), transaminitis (elevated AST), and acute kidney injury to rule out sepsis or metabolic disease (Table 1). On examination, he was severely malnourished, having lost 260 g from his birth weight, moderately dehydrated, irritable, and had a mildly distended abdomen with the liver palpable 2 cm below the costal margin. The rest of the examination was unremarkable.

**Table 1:** Comparison of laboratory and imaging findings between initial admission and readmission

Category	Parameter	Reference Range	Initial Admission	Interpretation	Readmission	Interpretation
Hematology	WBC	6–17 × 10³/µL	8.9 × 10³/µL	Normal	12 × 10³/µL	Normal
	Hemoglobin	10.5–14 g/dL	9.9 g/dL	Mild anemia	8 g/dL	Moderate anemia
	Platelet count	150–450 × 10³/µL	1093 × 10³/µL	Thrombocytosis	557 × 10³/µL	Mild thrombocytosis
Electrolytes and renal function	Sodium	135–145 mmol/L	154 mmol/L	Hypernatremia	151 mmol/L	Hypernatremia
	Potassium	3.5–5.5 mmol/L	2.6 mmol/L	Hypokalemia	5.6 mmol/L	Hyperkalemia
	Chloride	95–115 mmol/L	127 mmol/L	Hyperchloremia	120 mmol/L	Slightly elevated
	Creatinine	27–62 µmol/L	84 µmol/L	Mildly elevated	45 µmol/L	Normal
	Calcium	2.2–2.7 mmol/L	3.1 mmol/L	Hypercalcemia	2.3 mmol/L	Normal
	Magnesium	0.7–1.0 mmol/L	1.01 mmol/L	Mildly elevated	0.6 mmol/L	Hypomagnesemia
	Phosphorus	1.3–2.3 mmol/L	1.87 mmol/L	Normal	1.6 mmol/L	Normal
Acid-base status (ABG)	pH	7.35–7.45	7.14	Severe metabolic acidosis	7.34	Mild metabolic acidosis
	pCO₂	35–45 mmHg	24 mmHg	Respiratory compensation	26.7 mmHg	Respiratory compensation
	HCO₃⁻	18–24 mmol/L	8.3 mmol/L	Low bicarbonate	14.3 mmol/L	Low bicarbonate
Metabolic markers	Ammonia	11–35 µmol/L	86 µmol/L	Elevated	45 µmol/L	Normal
	Lactate	0.5–2.2 mmol/L	3 mmol/L	Mildly elevated	1.2 mmol/L	Normal
	Homocysteine	4–13 µmol/L	17 µmol/L	Elevated	Not repeated	—
	Glycine	100–300 µmol/L	Elevated	Likely antibiotic effect	Not repeated	—
Liver function tests	AST	20–60 IU/L	Elevated	Mild hepatocellular injury	Normal	Normal
	ALT	5–45 IU/L	Normal	Normal	Normal	Normal
	Bilirubin (Total)	0.3–1.0 mg/dL	Normal	Normal	Normal	Normal
Stool analysis	Reducing substance	Negative	Negative	Normal	Negative	Normal
	Stool electrolytes	K⁺ 15–30 mmol/24 h, Cl⁻ 10–20 mmol/24 h	K⁺ 2.1 mmol/24 h, Cl⁻ 5.8 mmol/24 h	Low K⁺, high Cl⁻	Not repeated	—
Urine test	Amino acid excretion	Normal	↑ Cysteine, ornithine, lysine, arginine	Suggestive of cystinuria	Not repeated	—
Immunology	IgA	7–40 mg/dL	—	—	83 mg/dL	Elevated
	IgG	200–900 mg/dL	—	—	1320 mg/dL	Elevated
	IgM	40–90 mg/dL	—	—	Normal	Normal
	IgE	<100 IU/mL	—	—	66 IU/mL	Normal
	Lymphocyte subsets	Normal	—	—	Normal	Normal
Microbiology	Blood culture	Sterile	No growth	Normal	Candida parapsilosis	Fungemia, cleared after treatment
	Urine culture	Sterile	No growth	Normal	>10⁵ cfu/mL *Enterobacter cloacae*	Urinary tract infections (UTI), repeated asymptomatic bacteriuria
	CSF PCR	Sterile	No growth	Normal	*E. coli* (initial), repeated negative	No CNS infection
Imaging	Renal ultrasound	Normal	Bilateral medullary nephrocalcinosis	Incidental finding	Stable nephrocalcinosis	Persistent
	Abdominal ultrasound	Normal	Unremarkable	Normal	Small free peritoneal fluid	Mild ascites
	Echocardiogram	Normal	No abnormality	Normal	Normal	No structural abnormality
Histopathology	Duodenal biopsy	Normal	Not done	—	Mild chronic nonspecific duodenitis	—
Genetic testing	Whole exome sequencing	—	Sent for evaluation	Workup initiated	SLC5A1 variant (glucose-galactose malabsorption)	Confirmed diagnosis
Other tests	Newborn screening	Normal	Normal	—	—	—

Initial investigations revealed normal hemoglobin and leukocyte levels but marked thrombocytosis. Electrolyte disturbance included hypernatremia, hyperchloremia, hypokalemia, hypercalcemia, and hypermagnesemia. Renal function appeared stable. Arterial blood gas analysis demonstrated severe metabolic acidosis. Both hyperammonemia and elevated lactate were noted. Liver function tests were largely unremarkable, except for raised AST. Stool studies were negative for reducing substances and cryptosporidium, and no infectious pathogens were identified. Stool electrolytes showed hypokalemia and hyperchloremia. A renal ultrasound incidentally revealed bilateral medullary nephrocalcinosis (Table 1).

IV fluid resuscitation led to partial improvement in electrolytes and acid-base balance. He was started on antibiotics, cefotaxime and vancomycin, which were later discontinued as blood, urine, and CSF cultures came back negative.

Due to the severity of presentation and persistent symptoms of diarrhea and abdominal distension, a variety of differential diagnoses were considered, and he was investigated accordingly for inborn errors of metabolism, malabsorption syndrome with chronic diarrhea, pseudohypoaldosteronism, renal tubular acidosis (type 4), and immunodeficiencies. Infectious causes and surgical causes were also ruled out. Urine analysis revealed increased excretion of cysteine, ornithine, lysine, and arginine, consistent with cystinuria. Plasma homocysteine and glycine levels were elevated, with the latter likely due to antibiotic therapy. Newborn screening results were normal (Table 1).

He was commenced on extensively hydrolyzed milk and then later on rice-based formula, on which diarrhea was improving. He was treated for cow milk protein allergy and was discharged in a stable state on rice-based formula milk.

After 48 hours, he was readmitted with progressively increasing abdominal distension. Laboratory tests once again showed electrolyte disturbance as hypernatremia, hyperkalemia, hyperchloremia, elevated creatinine levels, and persistent metabolic acidosis. Liver function remained within normal limits, though AST levels remained elevated. Immunoglobulin levels were also sent, which showed elevated IgA and IgG. Lymphocyte subsets were normal. Urine test was suggestive of urinary tract infection with *Enterobacter cloacae* complex; hence, he was started on trimethoprim-sulfamethoxazole. Abdominal ultrasound demonstrated stable nephrocalcinosis and small amounts of free peritoneal fluid (Table 1). His formula was changed back to extensively hydrolyzed milk, with improvement in abdominal distension; however, there was ongoing diarrhea. The patient underwent endoscopy, which revealed mild erythema in the lower esophagus and edematous mucosa in the stomach and duodenum. Biopsy findings indicated mild chronic nonspecific duodenitis. Meanwhile, a genetic test was also sent to identify the cause of his diarrhea.

He was eventually started on total parenteral nutrition, leading to an immediate resolution of diarrhea. However, after two weeks, he developed a fever and was investigated further. Blood cultures from both the central and peripheral lines grew *Candida parapsilosis*. Urine culture showed asymptomatic bacteriuria with *E. cloacae*. He was treated for fungemia caused by *C. parapsilosis* with antifungals: caspofungin for six days and fluconazole for 14 days. Additionally, cerebrospinal fluid (CSF) PCR detected *Escherichia coli*, though the child remained asymptomatic, and subsequent CSF analysis and cultures were negative. An echocardiogram revealed normal results, ruling out any cardiovascular abnormalities (Table 1).

TPN was discontinued, and he was put on an extensively hydrolyzed formula, to which he had intermittent diarrhea. Whole exome sequencing confirmed a pathogenic homozygous variant, c.83A>G P.(Asp28Gly), in the SLC5A1 gene, consistent with CGGM (Table 1). No association between primary immunodeficiency and glucose-galactose malabsorption (GGM) was reported; therefore, all infections that he had are considered to be secondary immunodeficiency due to severe malnutrition. The plan to start a fructose-based formula (galactomin 19) was hindered by its unavailability. Later, he was put on a protein- and fat-based (glucose-free, galactose-free, and lactose-free) formula in combination with an extensively hydrolyzed whey-based formula, which he tolerated well. His stools have since become non-watery, indicating significant clinical improvement. He was discharged in a stable state. On follow-up, he was found to be thriving well on fructose-based formula with his weight in the 19th centile at the age of five months.

## Discussion

CGGM is a rare autosomal recessive disorder caused by mutations in the SLC5A1 gene, located on chromosome 22q13.1. This gene encodes the sodium-glucose co-transporter 1 (SGLT1), which is primarily found on the brush border membrane of intestinal epithelial cells and, to a lesser extent, in the renal tubules [1,2]. SGLT1 plays a crucial role in the absorption of glucose, amino acids, vitamins, and various ions across the intestinal lining [3].

To date, more than 300 cases of CGGM have been reported worldwide, with over 40 mutations identified in the SLC5A1 gene [4,5]. Since CGGM follows an autosomal recessive inheritance pattern, parental consanguinity poses a significant risk factor. A literature review (2001-2019) by Xin and Wang analyzed 107 cases of CGGM, revealing that among 55 cases from Saudi Arabia and Turkey, 43 (78.2%) were born to consanguineous parents. The parents were consanguineous in our patient’s case as well.

A defect in SGLT1 results in the accumulation of unabsorbed glucose, galactose, and sodium in the intestinal lumen, creating an osmotic gradient that draws water into the intestines. This leads to severe osmotic diarrhea, the hallmark manifestation of CGGM. Apart from the severe watery diarrhea beginning from neonatal onset, affected infants also present with dehydration, failure to thrive, abdominal distension, electrolyte imbalances, and colic-like irritability [6]. Hypernatremic dehydration is seen to be a common feature occurring due to excessive water losses precipitated by the osmotic diarrhea. The frequency of hypernatremic dehydration in children with GGM has been reported to be 25% [7].

Other manifestations of CGGM, such as bilateral renal stones and renal tubular acidosis (RTA), have been reported in CGGM patients [8-10]. Since the SGLT1 gene is expressed in renal tubular cells, its mutation may lead to mild impairment of the renal glucose transport. Some CGGM patients develop glucosuria, which may also increase the risk of urinary tract infections (UTIs) and kidney stone formation [7]. In our patient, renal ultrasound revealed bilateral medullary nephrocalcinosis, and urine culture showed asymptomatic bacteriuria with *E. cloacae*.

Another diagnostic feature of CGGM is a stool pH of less than 5.3, resulting from the fermentation of undigested glucose and galactose. This process generates short-chain fatty acids, leading to acidic stools. Additionally, stool-reducing sugars are positive, and the stool osmotic gap is typically greater than 40 mOsm [11]. Therefore, screening the stool for acidic pH and reducing substances can be used as diagnostic tools. However, in our patient, the stool-reducing substance came back negative.

A biopsy of the small intestine with normal histopathology can rule out other causes of malabsorption. Biopsy findings in our patient only indicated mild chronic nonspecific duodenitis. A key factor that played in the diagnostic approach for our patient was the elimination of enteral feeding, which resulted in the cessation of diarrhea. Different formulas were tested initially, such as rice-based formula and extensively hydrolyzed formula, which only led to mild improvement in the diarrhea, whereas starting the patient on total parenteral nutrition led to the complete resolution of the diarrhea.

Ultimately, genetic testing (WES) confirmed the diagnosis of CGGM. A fructose-based formula was thereafter introduced, which did not cause any diarrhea for our patient. This is explained by the mechanism of fructose absorption in the small intestine, which is facilitated by a distinct transporter (GLUT5/SLC2A5); therefore, it remains unaffected in CGGM [11].

## Conclusions

GGM should be considered in infants with persistent diarrhea and failure to thrive, as timely diagnosis optimizes prognosis, improves outcomes, and facilitates genetic counseling for affected families. The patient is being followed by the gastroenterology team and the nutrition department on an outpatient basis, and he is thriving well with no symptoms while on fructose-based formula (galactomin 19).

CGGM is a rare condition that should not be overlooked when managing a neonate with chronic diarrhea after the most common causes have been ruled out. Genetic testing should be considered in the diagnostic workup at an earlier stage in suspected patients to prevent serious complications and improve prognosis. It is also important to highlight that parents should be provided with ongoing support and education on managing their child’s diet to help facilitate adequate nutrition for healthy growth.
